# Supplementary figures and images for: Alternative splicing in pediatric central nervous system tumors highlights oncofetal candidate CLK1 exon 4
Source: Neurooncol Pediatr. 2026 Feb 16;2(1):wuag009. doi: 10.1093/neuped/wuag009 (PMC13017157; doi:10.1093/neuped/wuag009)

Figure S1

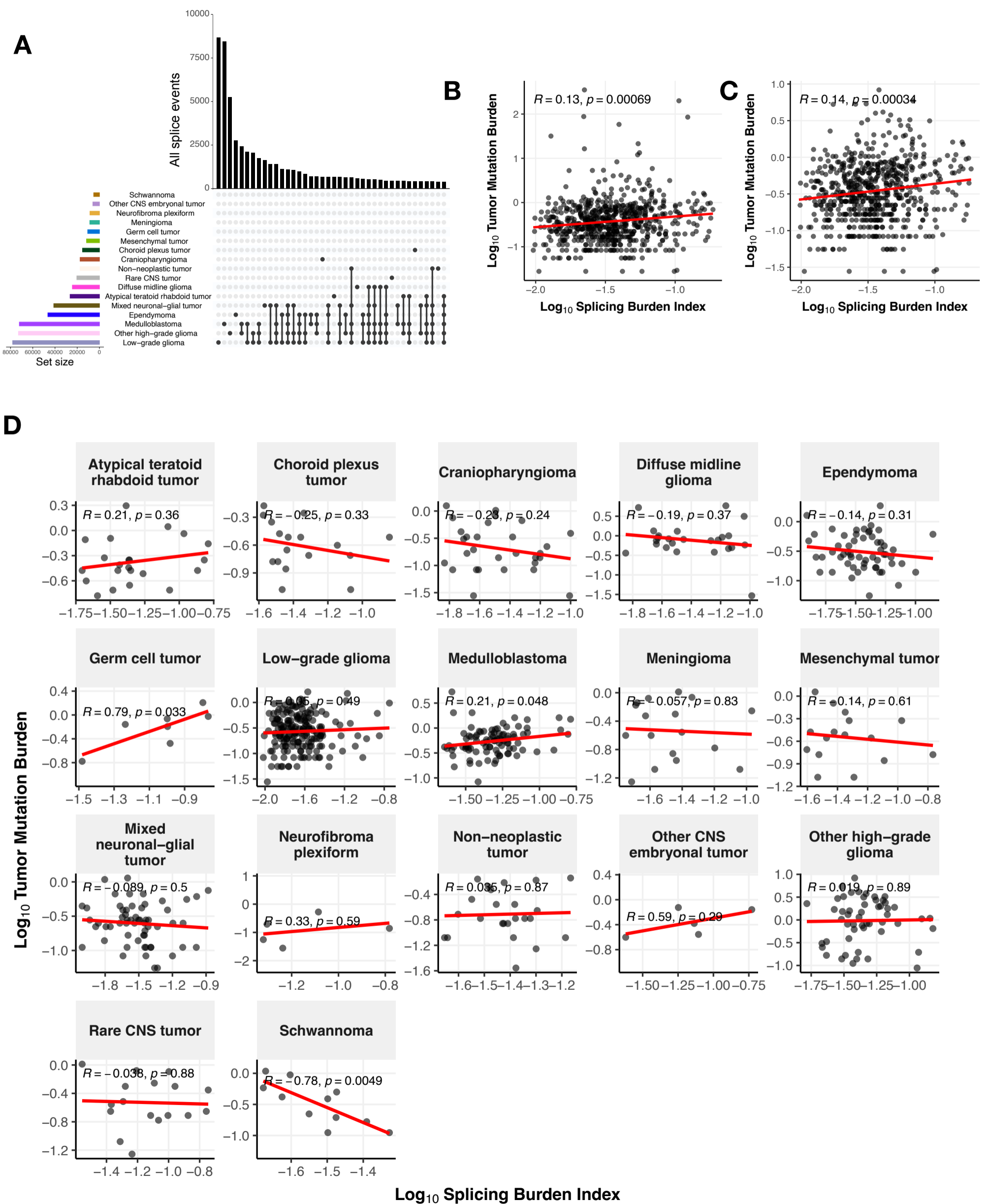

Figure S2

A

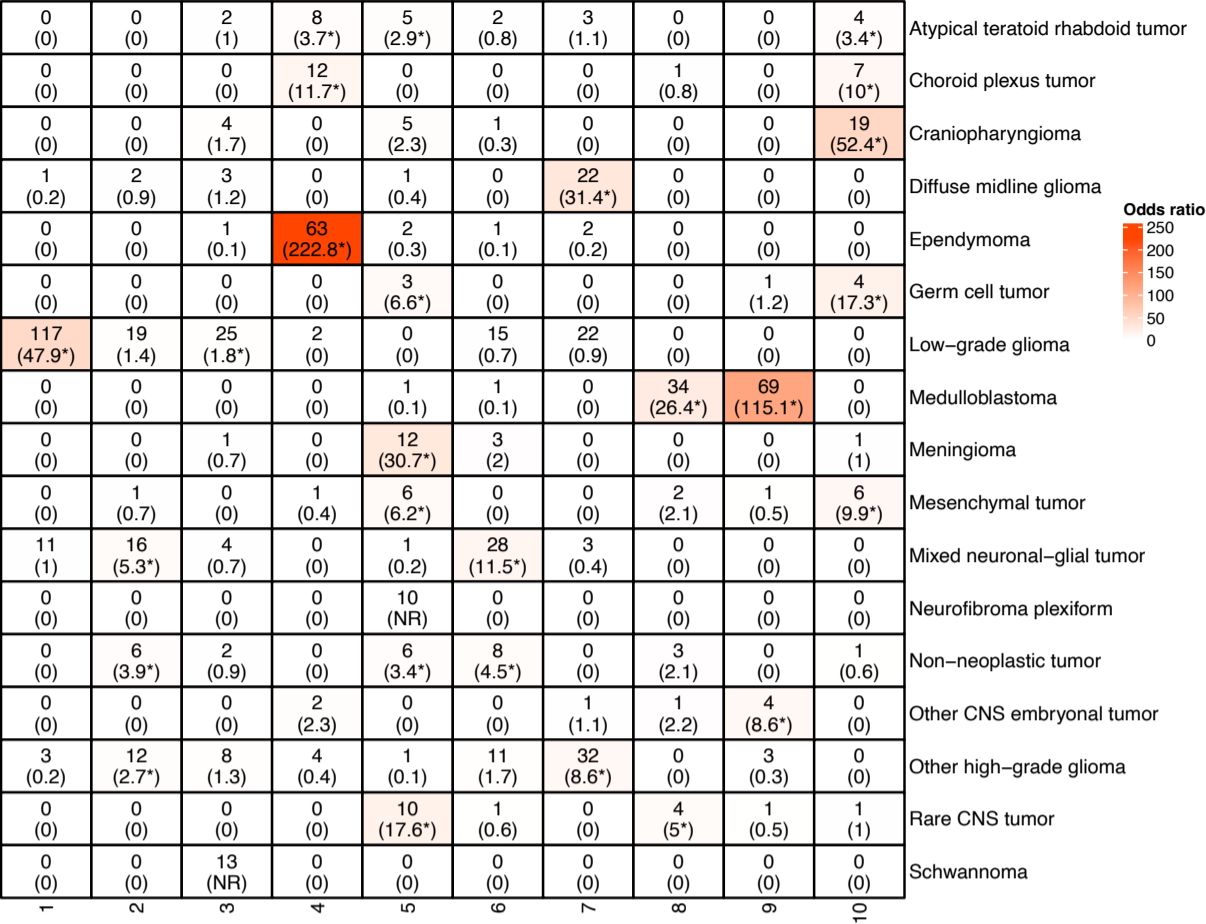

B

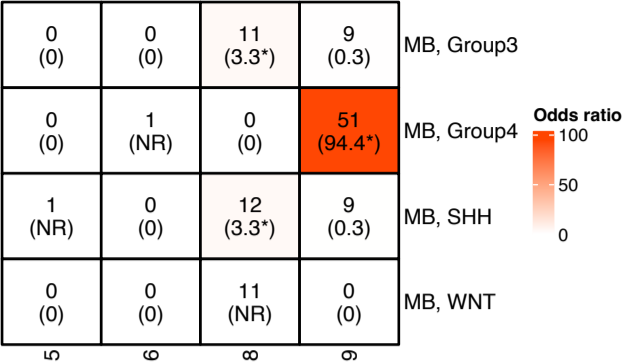

C

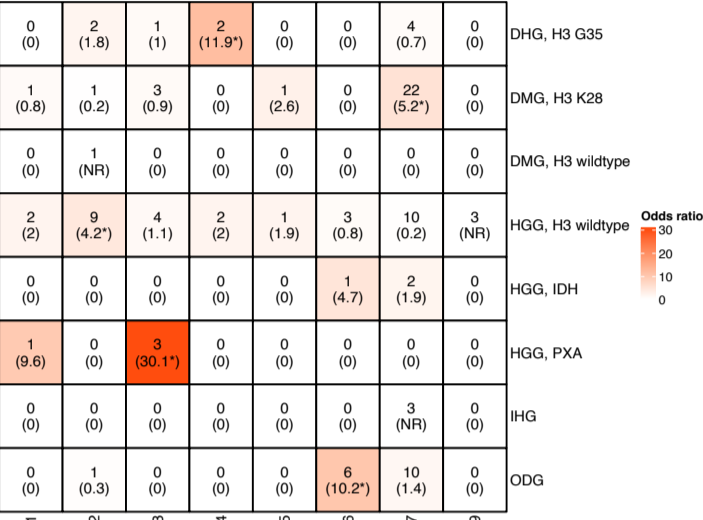

D

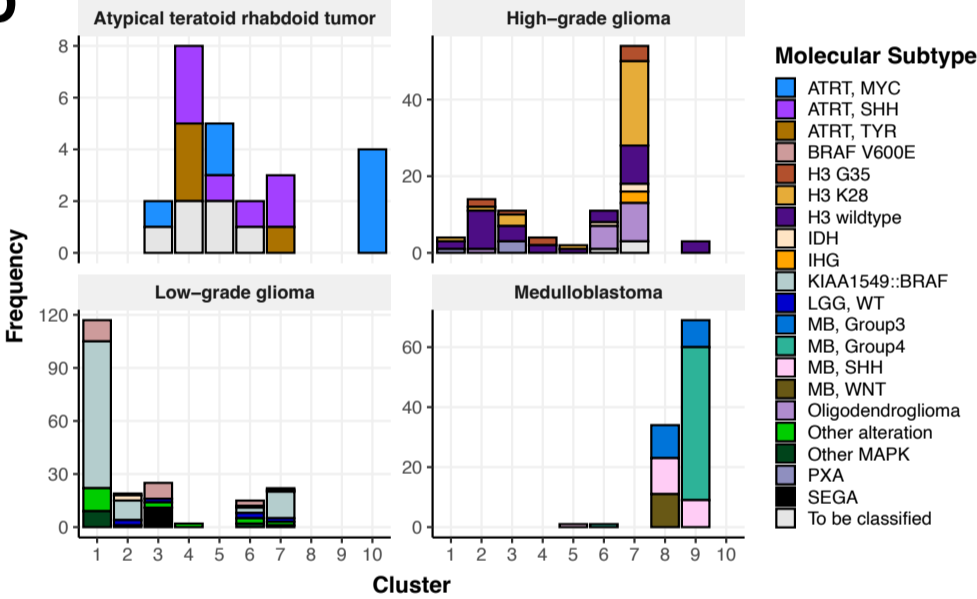

E

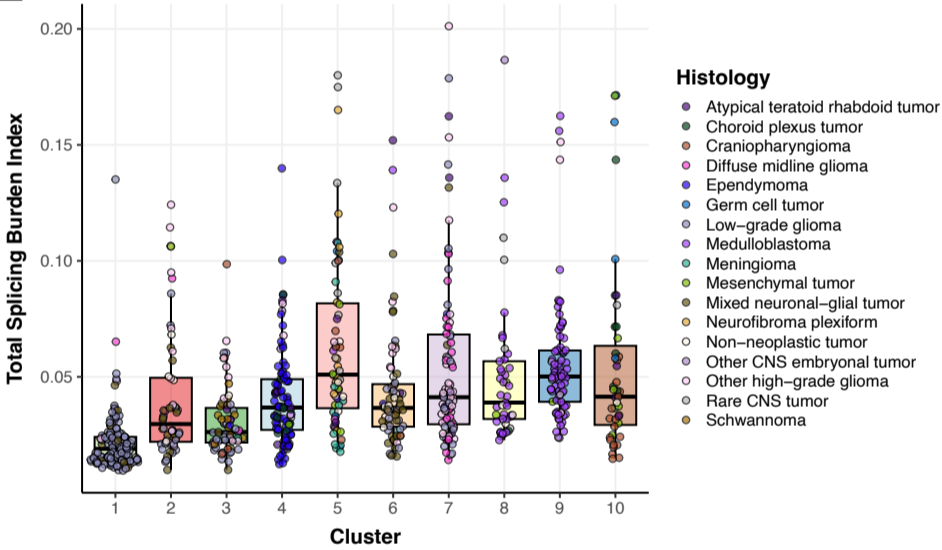

Figure S3

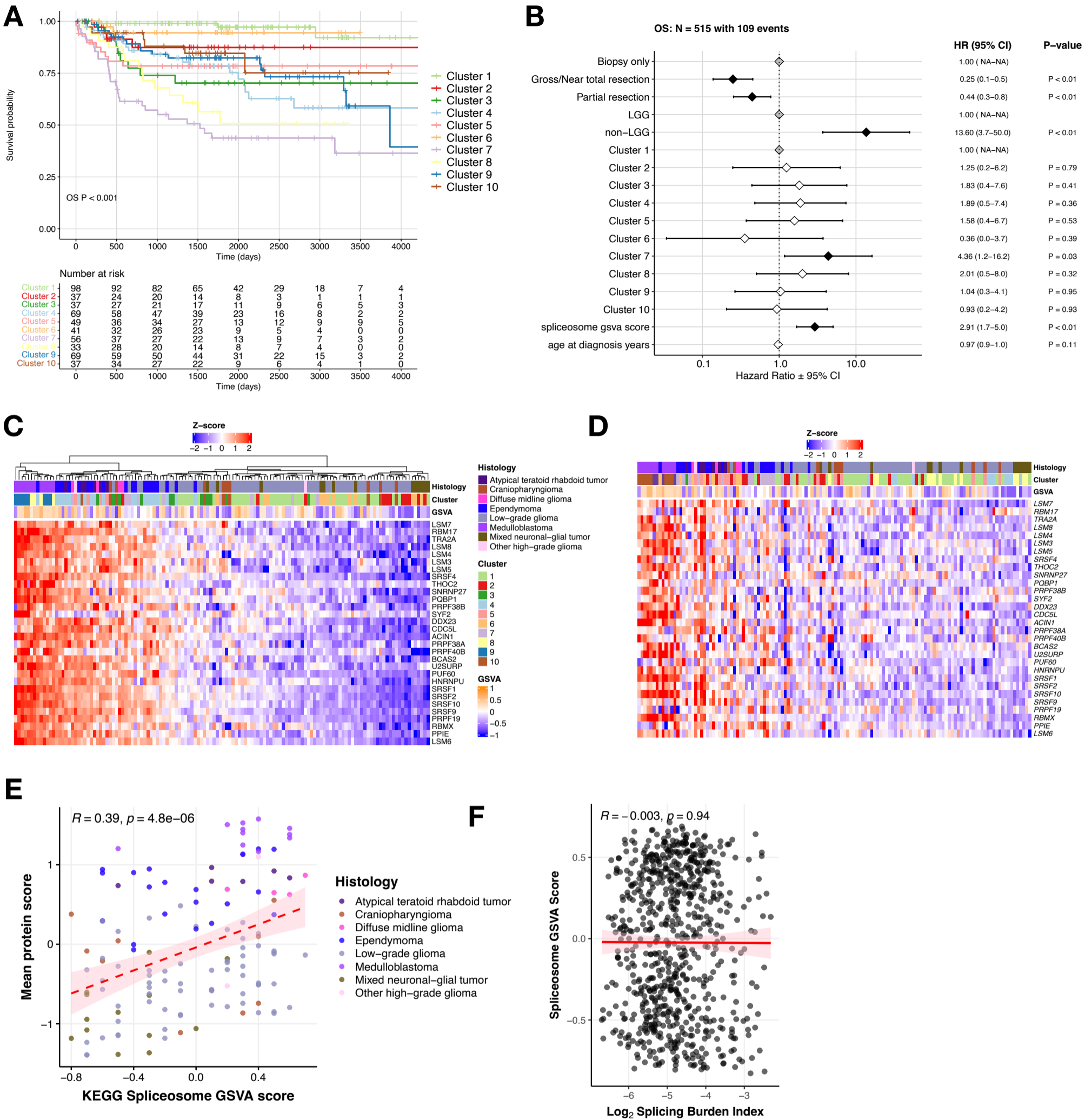

Figure S4

A

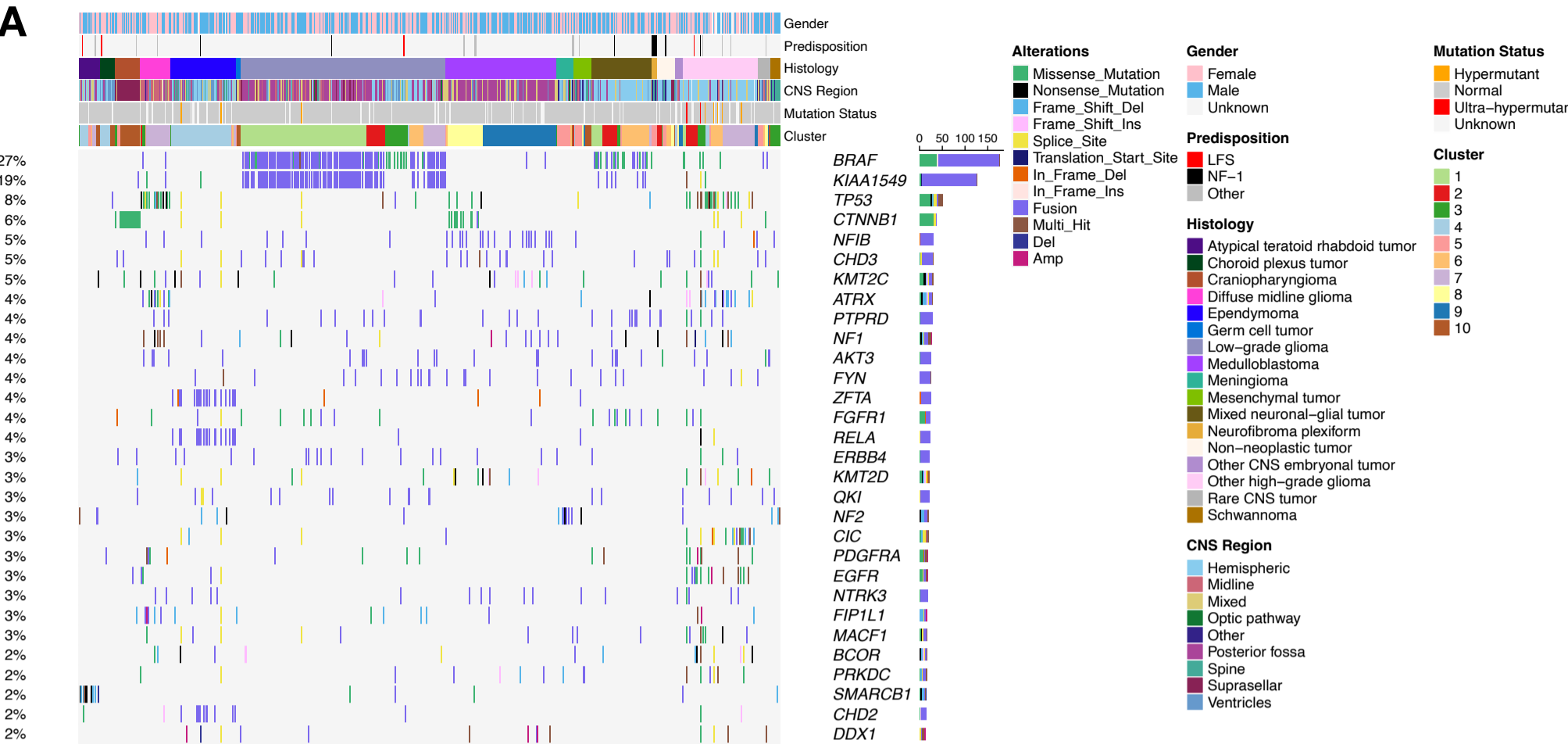

B

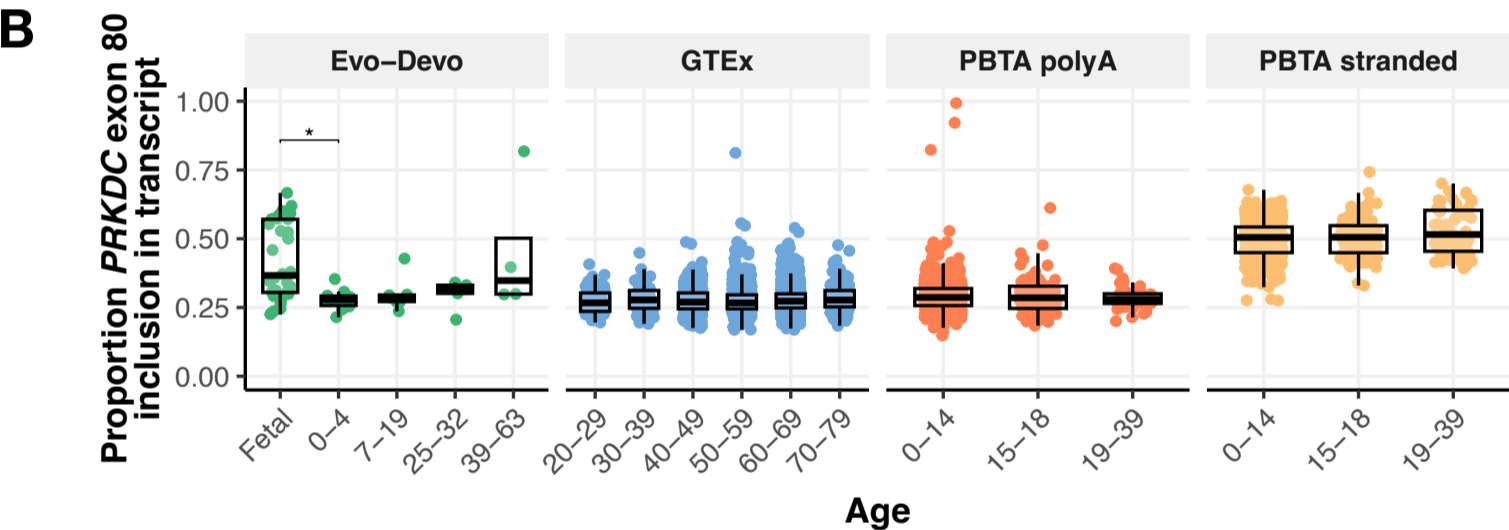

C

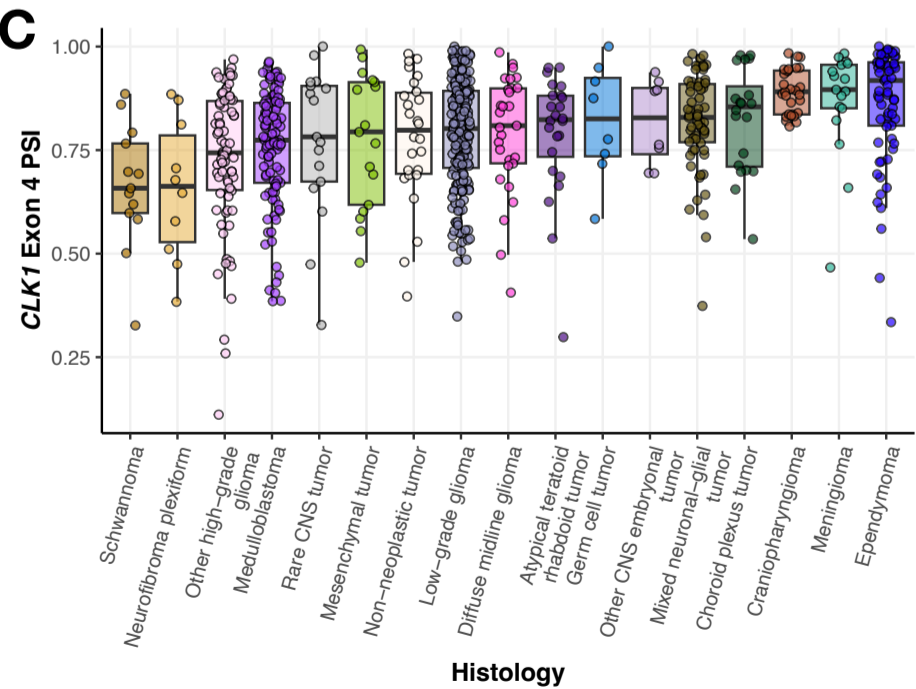

D

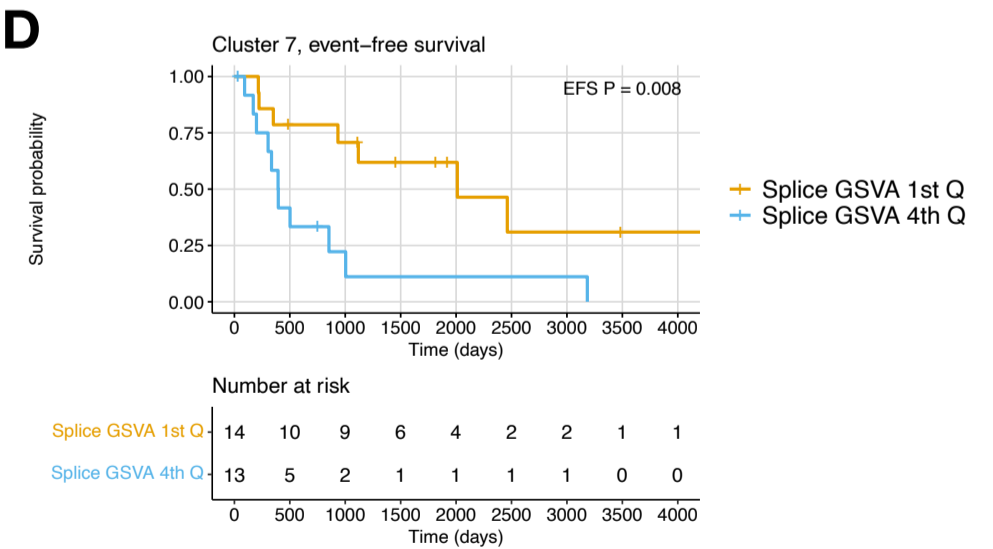

Figure S5

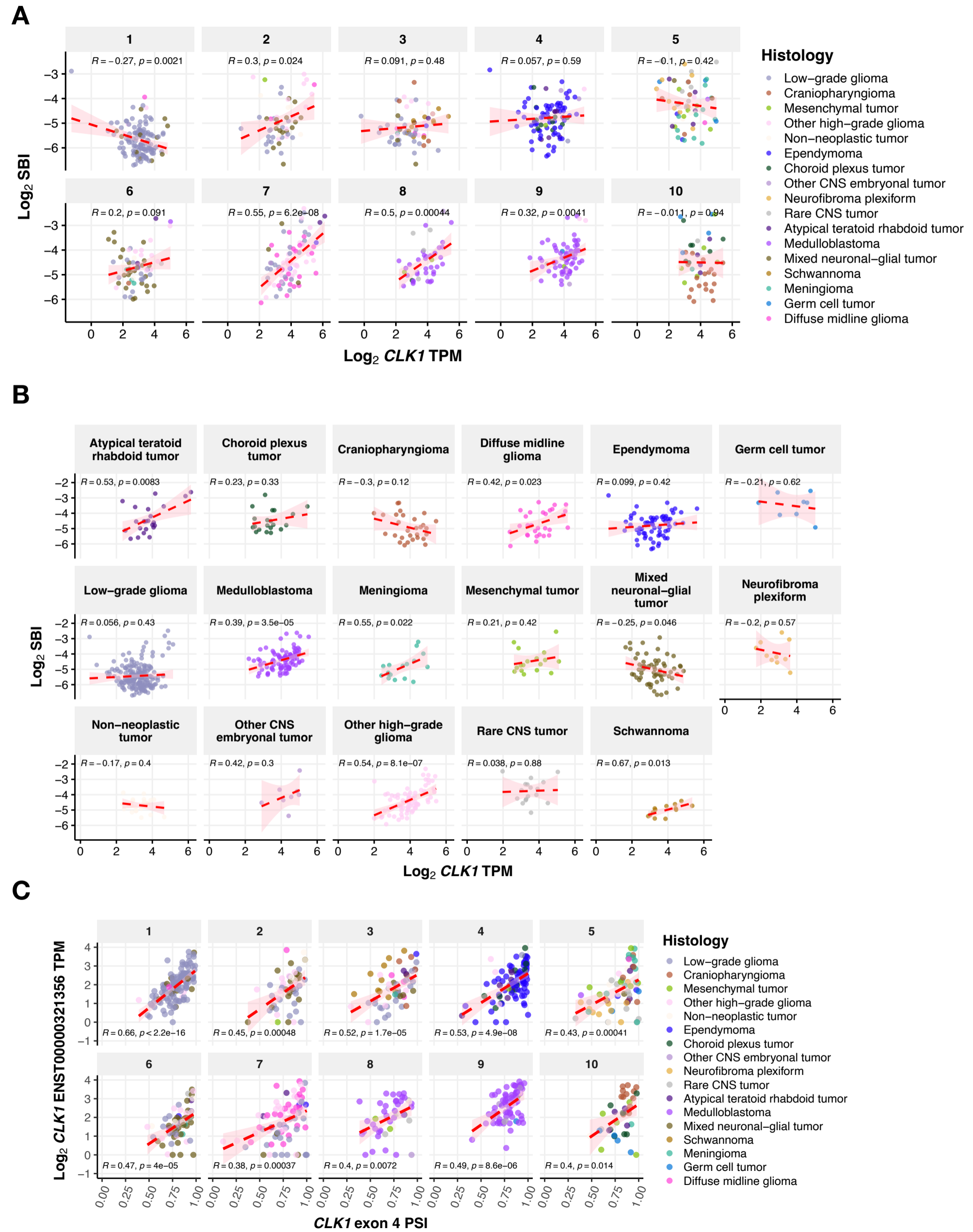

Figure S6

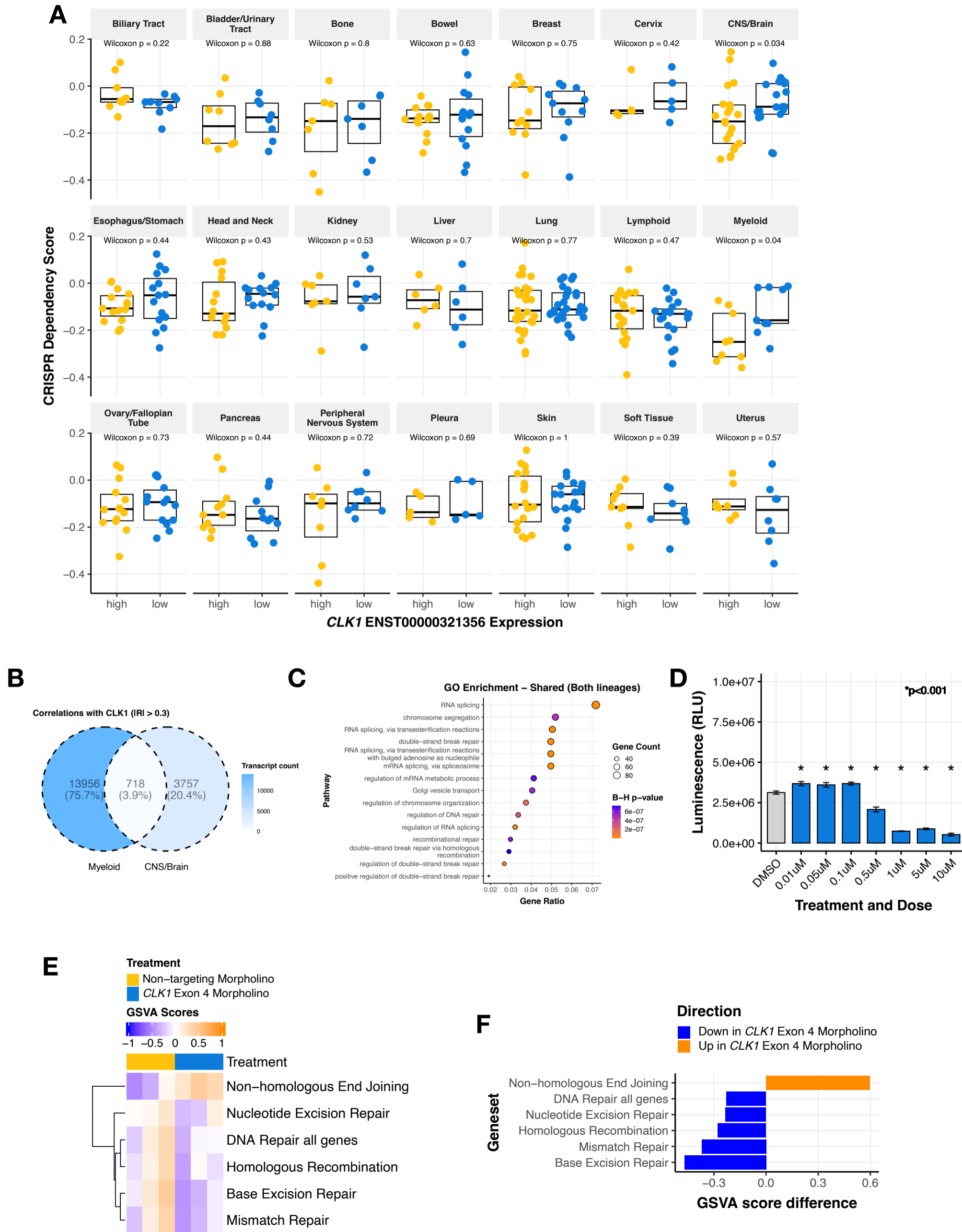

Supplement: wuag009_Supplementary_Data [file wuag009_supplementary_data.zip › Supplemental-Figures.pdf]
